# Identification and Classification of New Transcripts in Dorper and Small-Tailed Han Sheep Skeletal Muscle Transcriptomes

**DOI:** 10.1371/journal.pone.0159638

**Published:** 2016-07-19

**Authors:** Tianle Chao, Guizhi Wang, Jianmin Wang, Zhaohua Liu, Zhibin Ji, Lei Hou, Chunlan Zhang

**Affiliations:** 1 Shandong Provincial Key Laboratory of Animal Biotechnology and Disease Control and Prevention, College of Animal Science and Veterinary Medicine, Shandong Agricultural University, Taian 271018, China; 2 College of Biological and Agricultural Engineering, Weifang University, Key Laboratory of Biochemistry and Molecular Biology in Universities of Shandong, Weifang 261061, China; Wageningen UR Livestock Research, NETHERLANDS

## Abstract

High-throughput mRNA sequencing enables the discovery of new transcripts and additional parts of incompletely annotated transcripts. Compared with the human and cow genomes, the reference annotation level of the sheep genome is still low. An investigation of new transcripts in sheep skeletal muscle will improve our understanding of muscle development. Therefore, applying high-throughput sequencing, two cDNA libraries from the biceps brachii of small-tailed Han sheep and Dorper sheep were constructed, and whole-transcriptome analysis was performed to determine the unknown transcript catalogue of this tissue. In this study, 40,129 transcripts were finally mapped to the sheep genome. Among them, 3,467 transcripts were determined to be unannotated in the current reference sheep genome and were defined as new transcripts. Based on protein-coding capacity prediction and comparative analysis of sequence similarity, 246 transcripts were classified as portions of unannotated genes or incompletely annotated genes. Another 1,520 transcripts were predicted with high confidence to be long non-coding RNAs. Our analysis also revealed 334 new transcripts that displayed specific expression in ruminants and uncovered a number of new transcripts without intergenus homology but with specific expression in sheep skeletal muscle. The results confirmed a complex transcript pattern of coding and non-coding RNA in sheep skeletal muscle. This study provided important information concerning the sheep genome and transcriptome annotation, which could provide a basis for further study.

## Introduction

As one of the most important meat production animals worldwide, sheep have always held an important position in animal husbandry. Therefore, enhancing the understanding of the regulatory mechanism of muscle growth in sheep and identifying genes that regulate the growth of skeletal muscle are of great significance. However, compared to the more comprehensively studied human [1], mouse [2], maize [3] and cow [4] transcriptomes, sheep transcriptomic research remains at a low level. Recently, with the development of whole-transcriptome sequencing, several studies have been undertaken to research the regulation in various specific sheep tissues in diverse researches [5–11]. However, to our knowledge, most of these high-throughput mRNA sequencing (RNAseq) studies have neglected to explore the unannotated transcripts in sheep.

RNAseq makes it possible to reveal the expression profiles underlying phenotype, metabolic and physiological changes and different stages of development and environmental impacts at single-base resolution [4]. Another important capacity of RNAseq is the revelation of unannotated transcriptional activity. By identifying massive novel transcripts, we can find new gene loci (protein coding and noncoding) and can complement the structure of the known genes [12–14].

In our previous study, to obtain an accurate map of annotated transcripts together with their expression in sheep skeletal muscle, high-throughput sequencing was applied to construct two cDNA libraries from the biceps brachii of small-tailed Han (SH) sheep and Dorper (DP) sheep, and whole-transcriptome analysis was performed to describe the comprehensive transcript catalogue of this tissue [15,16]. Dorper sheep are a South Africa breed with good muscle conformation to produce a desirable carcass. They have a flat back and wide waist, and their legs are short and thin. While the small-tailed Han sheep, with long, strong limbs and an elliptical fanshaped tail, are indigenous breed in China that provide good flavored meat and are rich in fat. Dorper sheep are famous as a meat-producing breed for their rapid muscle growth, while small-tailed Han sheep have a higher mean litter size (2.61) [17] than Dorper sheep (1.45 to 1.60) [18]. The results of our previous study were analyzed to report differentially expressed genes, alternative splicing, coding single-nucleotide polymorphisms (cSNPs) and new transcription units [15,16]. However, the dataset of unannotated new transcripts still lacks accurate identification and annotation. Furthermore, recent updates to the sheep genome and gene annotation have reduced the reference value of the results of the original new transcript analysis, which not only led to wasted data but also hindered the in-depth understanding of the regulation of skeletal muscle. Accordingly, we decided to reanalyze the sequencing data to find new transcripts. The functions of these transcripts are largely unknown, although there is increasing evidence that new transcripts play key roles across diverse biological processes, with an emerging theme of interfacing with epigenetic regulatory pathways [19, 20]. Thus, the sheer number and increasing pace of discovery of new transcripts are accompanied by the growing challenge of their definition and annotation [21]. We believe that these newly identified transcripts lacking annotation information in the reference sheep genome might play important roles in the complex regulatory processes of sheep skeletal muscle.

In this study, to describe the comprehensive transcript catalogue of sheep skeletal muscle, we focused on the identification of new transcripts expressed in this tissue, including protein-coding RNA and non-coding RNA. Using RNAseq and bioinformatics analyses, a group of new transcripts was identified and underwent detailed classification. This study of new transcripts will provide important information concerning the sheep genome and transcriptome annotation and will provide a basis for our future work on sheep skeletal muscle.

## Materials and Methods

### Ethics Statement

All animal experiments were approved by the Institutional Animal Care and Use Ethics Committee of Shandong Agricultural University (Permit Number:2004006) and performed in accordance with the “Guidelines for Experimental Animals” of the Ministry of Science and Technology (Beijing, China). All surgery was performed according to recommendations proposed by the European Commission (1997), and all efforts were made to minimize suffering.

### Sampling, library preparation and sequencing

Healthy 11-month-old Dorper and small-tailed Han ewes were obtained from the Linqv Huanong Sheep Farm (Weifang, Shandong, China). The appearance and shape of the sheep completely conformed to their varietal characteristics [22]. The selected sheep are healthy individuals with moderate weight. All of the sheep were raised under the same conditions of free access to water and food in natural lighting. The fresh biceps brachii tissue samples from the sheep were collected immediately after a quickly slaughter, cut into pieces of 3g and quickly placed into liquid nitrogen, and then the tissue blocks were stored at -80°C for long term preservation until use. More details of animals selection, biceps brachii collection, construction of cDNA libraries and sequencing were described as previous reports [15]. The deep sequencing data obtained were deposited in the GEO database with the accession number GSE43316.

### Mapping and annotation

Using Bowtie [23], SAMtools [24] and TopHat [25], we mapped our reads to the sheep reference genome v3.1 (ftp://ftp.ncbi.nlm.nih.gov/genomes/Ovis_aries/). Assembled reads were annotated with the NCBI reference annotation (release 101, ftp://ftp.ncbi.nlm.nih.gov/genomes/Ovis_aries/GFF/) using Cufflinks [26]. The resulting individual transcripts were merged to form a single transcript assembly with Cuffmerge. The merged transcript was applied for locus and transcript quantification using Cuffdiff. To obtain the unannotated transcripts, the known transcripts were filtered out with Cuffcompare. Transcripts with class code u were detected as unannotated new transcripts.

### Protein coding potential prediction

The Coding Potential Calculator (CPC, version 0.9-r2) [27] was applied to distinguish coding and non-coding transcripts from our datasets. To achieve higher reliability for the coding potential prediction, two protein databases (UniRef90 and NCBI nr) were applied separately with CPC prediction. Furthermore, to reduce the arbitrariness of the threshold for this program, the same cut-off values used by R Weikard et al [4] were adopted as our threshold.

### Comparative sequence analysis

To identify transcripts that were already annotated in other species or related to known genes, BLASTN (v2.2.26+, e-value = 1e-10) and several manual approaches were applied for comparative sequence analysis.

First, using BLASTN, we compared our transcripts to the NCBI Refseq database (human, cow and sheep), UTRdb [28] (human only) and Gnomon (sheep only) to find highly similar sequences. The criteria for comparative analysis were defined as follows: sequence mapping identity ≥75% in a covered region ≥100 nt for human, sequence mapping identity ≥90% in a covered region ≥100 nt for cow, and sequence mapping identity ≥95% in a covered region ≥100 nt for sheep.

The comparison results were then manually processed for detailed classification. For this purpose, transcripts with accepted hits were further analyzed to examine the information on current annotation status, exon structure, position on chromosome, nearest neighbor gene and protein coding potential. The results of sequence similarity analysis could only be accepted when the new transcripts showed similar structures and were located on orthologous chromosome areas between the two species with acceptable BLAST results.

### LncRNA identification

The identification of long non-coding RNA (lncRNA) was performed using the lncRNA identification pipeline, lncRNA Finder [3], which was released in the GitHub Repository (https://github.com/caulilin/lncRNA_Finder), to detect different types of lncRNAs. The putative lncRNA transcripts were further analyzed as described below.

In the first step, transcripts smaller than 200 nt were excluded from our dataset. Then, all transcripts encoding complete open reading frames (ORFs) of more than 100 aa were excluded from our dataset. The remaining transcripts were aligned to the Swiss-Model database [29] to eliminate transcripts with potential protein-coding capacity (cut-off E-value < = 0.001). Finally, to rule out housekeeping lncRNAs and microRNA (miRNA) precursors, putative lncRNAs were aligned to housekeeping lncRNA datasets, including the tRNA datasets downloaded from the Genomic tRNA Database (http://gtrnadb.ucsc.edu/download.html), rRNA collected from GenBank, and miRNA datasets downloaded from miRBase (http://www.mirbase.org/cgi-bin/mirna_summary.pl?org=oar) [30]. Potential lncRNAs that showed significant (e-value = 1e-10) mapping identity with housekeeping lncRNAs and miRNAs were filtered out from our dataset of lncRNA. Potential lncRNAs showing no significant alignment with miRNA were detected with high confidence as lncRNA.

### Validation of transcripts using RT-PCR

The RT-PCR primers used for validation were designed according to the sequences of selected identified transcripts using Primer Premier 5.0 (Premier, Canada). Primer sequences are given in Additional file 1. The cDNA was synthesized by using Primerscript RT reagent Kit (TaKaRa) with 1 μg of total RNA. The PCR amplification was carried out using 1.5μl of cDNA as the template, 12.5 μl of 2× PCR MasterMix (Tiangen), 0.5 μl primers (0.5 μmol each), and RNase-free dH_2_O to a final volume of 25 μl. After a 5 min denaturation at 94°C, PCR was performed for 30 cycles. Each cycle consisted of 94°C for 30 sec, 60°C for 30 sec, and 72°C for 30 sec, followed by a 72°C elongation for 5 min and 5 μl of each PCR product was analyzed using a 1% agarose gel. The primers of the housekeeping gene GAPDH used for the loading control are described in (S1 Table).

## Results

### Mapping and annotation of the transcripts identified in sheep skeletal muscle

After mapping, annotating and sorting reads, more than half (69.3 million) of the total reads (103.1 million) could be mapped to the reference sheep genome (DP, with 69.38% alignment, higher than SH, with 65.07%). Over half of the total reads (DP, with 53.65% alignment, lower than SH, with 56.34%) could be mapped to the reference genes (14,124 gene loci in DP and 13,928 gene loci in SH), while the unannotated genome region mapped reads were assembled into new transcripts (8,064 transcripts in DP, 6,368 transcripts in SH). Compared with our previous report [15], the genome matching rate was slightly decreased while the reference gene matching rate and gene loci number were increased (S1 Fig).

After transcript merging, 40,129 co-expressed unique primary muscle transcripts were ultimately assigned to the sheep genome. A total of 36,662 transcripts (91.36%) were mapped to 12,918 known gene loci annotated in the reference sheep genome. Among them, 22,008 (54.84%) transcripts were classified as known transcripts of annotated genes, and 14,654 transcripts (36.52%) were assigned to known transcript regions. A total of 3,467 transcripts (8.64%) had not been annotated in the reference sheep genome. In this study, we focused our attention on the identification and classification of these unannotated new transcripts, as transcripts with clear annotations were not the focus of this study. For this purpose, the dataset of 3,467 new transcripts (S2 Table) without annotation was adopted for further analysis.

**Fig 1:**
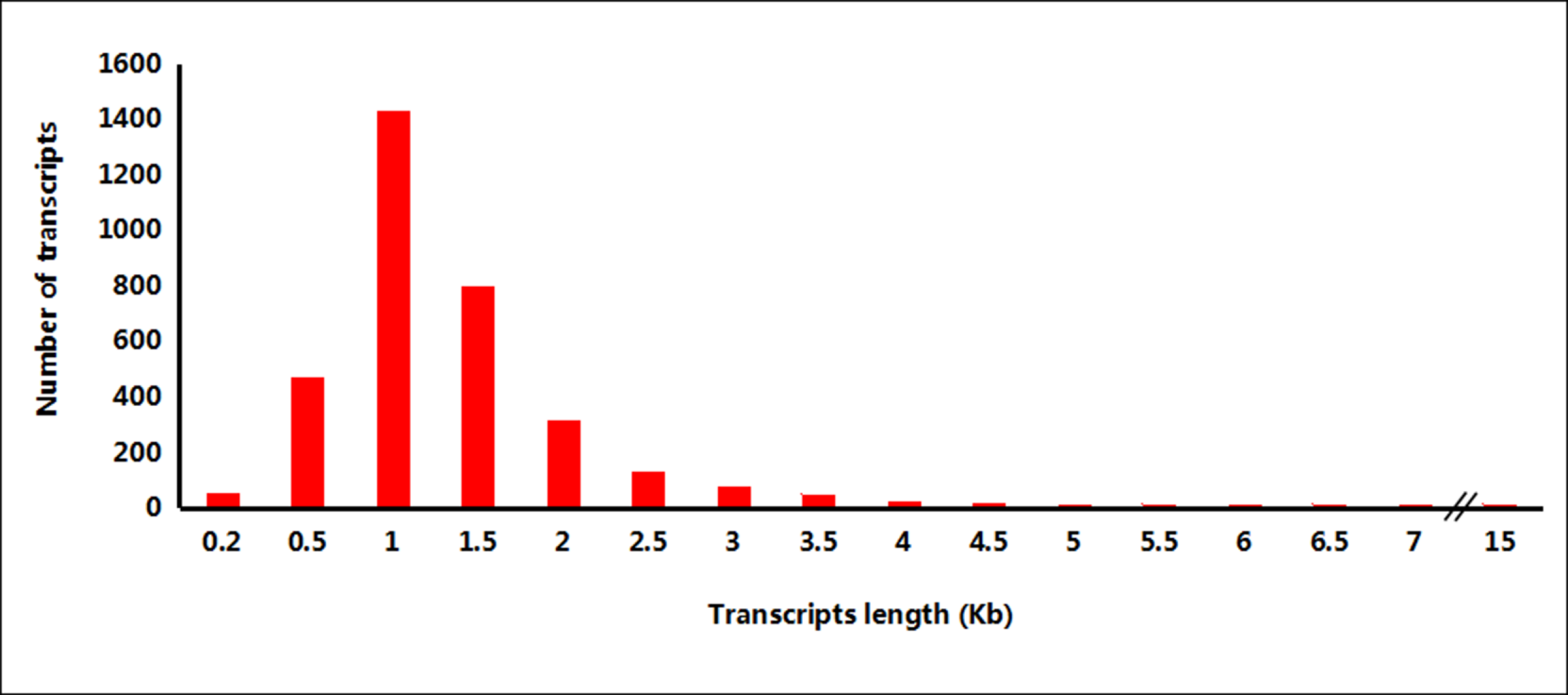
Length variation and number of new transcripts detected in sheep biceps brachii.

**Fig 2:**
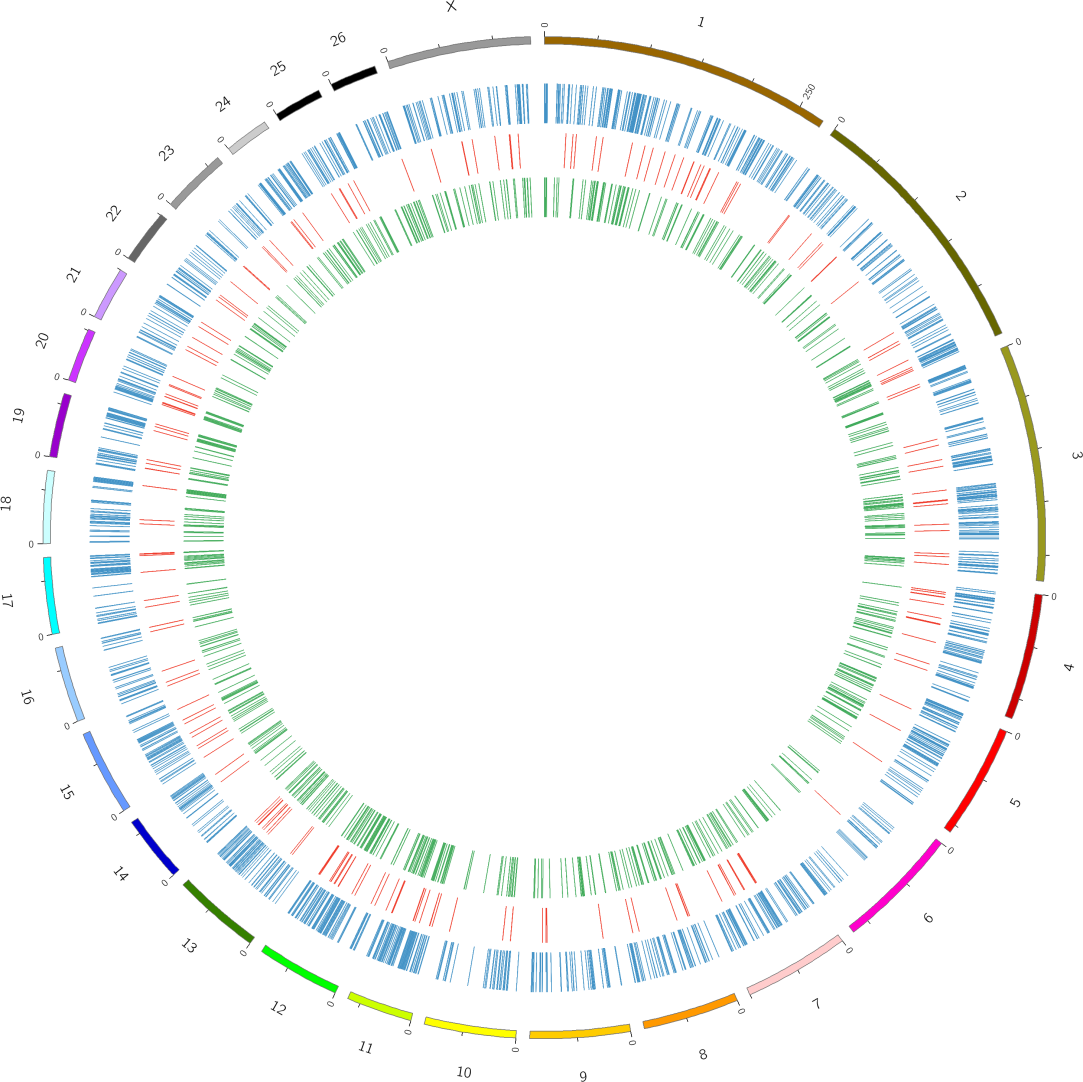
Intuitive map of new transcripts on sheep chromosomes. Chromosomes are shown in different colors in the outermost circle, and the innermost circles show the distribution of each transcript. Blue: Distribution of all new transcripts. Red: Distribution of transcripts with comparative sequence analysis results. Green: Distribution of lncRNA transcripts.

**Fig 3:**
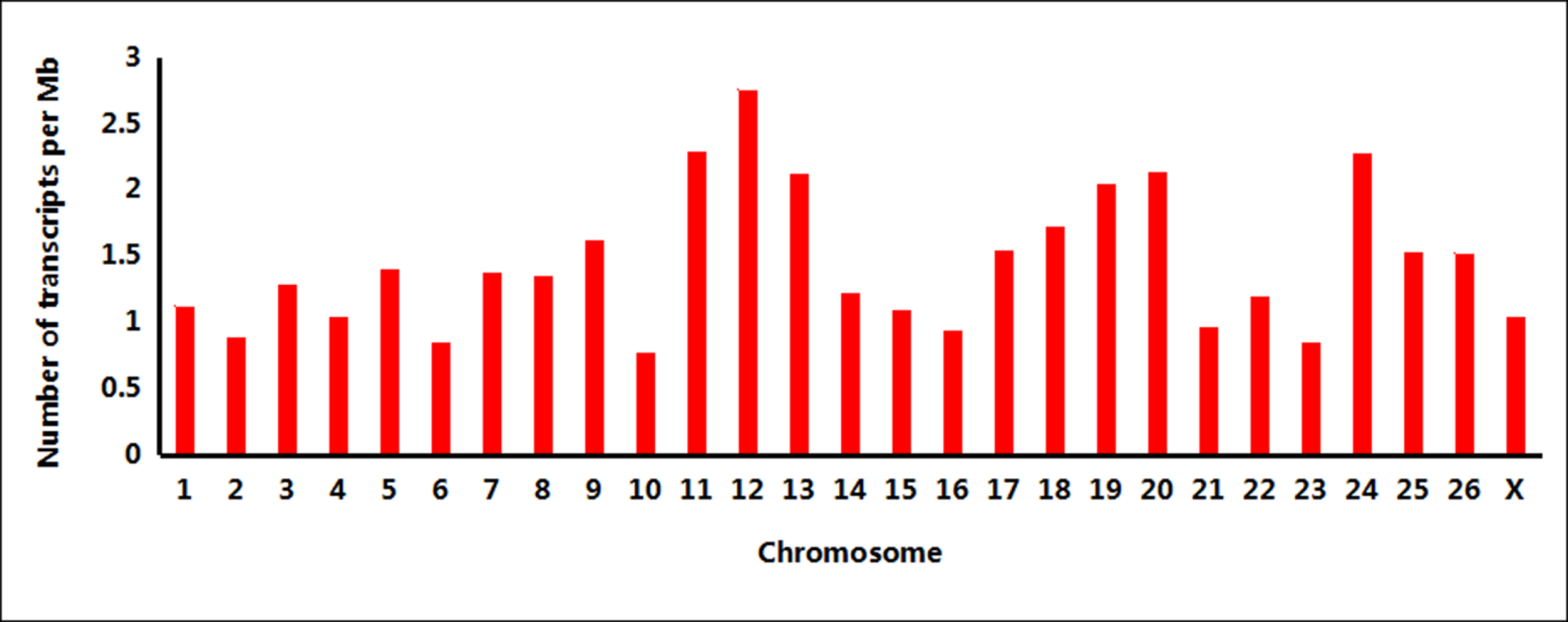
Number of new transcripts per Mb per chromosome. Distribution density of new transcripts on 26 sheep autosomes and the X allosome are shown.

Among the new transcripts, only 203 transcripts show multiple exon structures (from 2 to 23), while the other 3,271 transcripts consist of single exons. The sizes of the new transcripts mapped in the reference sheep genome ranged from 92 to 14,775 bp, with an average length of 1172 bp, which is much higher than the average length of 343 bp in our previous report [16]. Most of these new transcripts had sizes distributed between 500 bp and 2 kb (Fig 1). The highest numbers of new transcripts were detected on sheep chromosomes 1 and 3, which are also the longest chromosomes (Fig 2). However, the densest transcript distribution areas were on chromosomes 11 and 12, which may indicate that the new transcripts were not uniformly distributed among the chromosomes (Fig 3).

**Fig 4:**
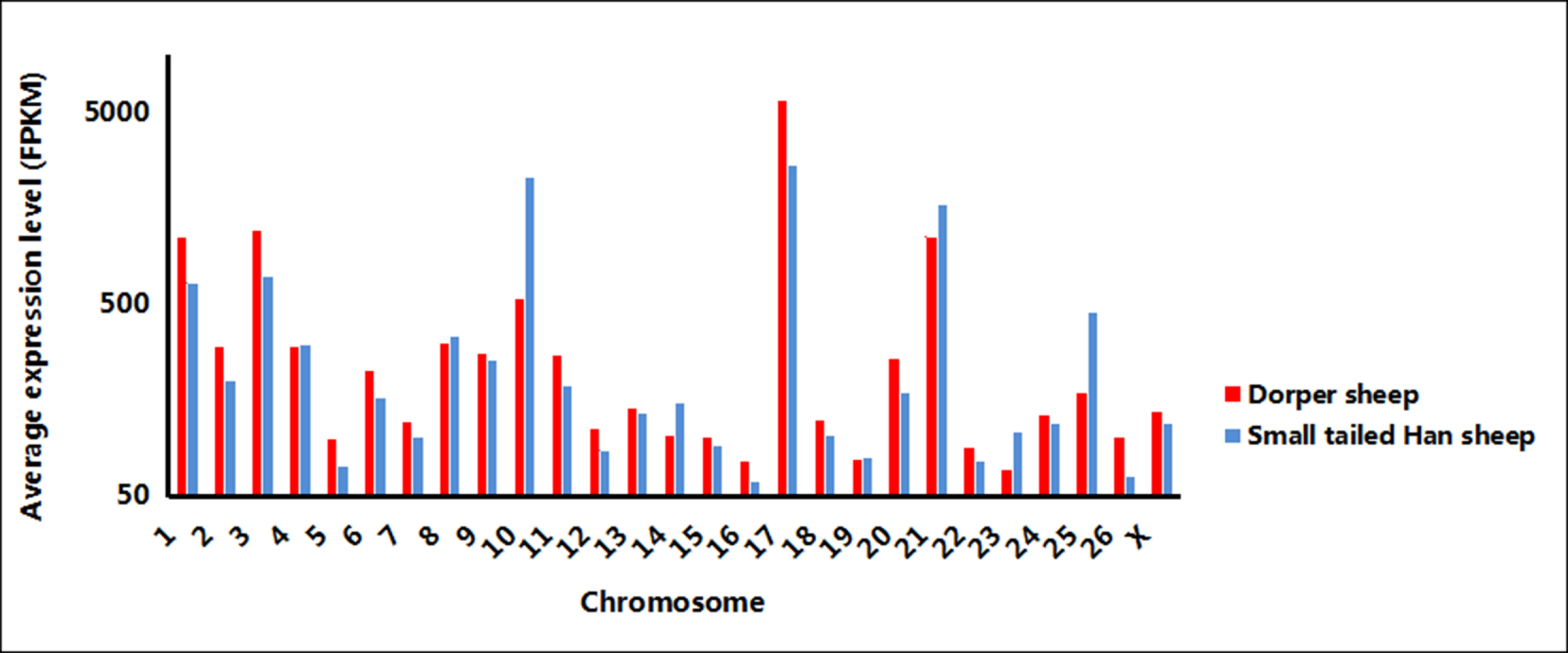
Average expression level per chromosome of new transcripts in Dorper sheep and small-tailed Han sheep. Red: Dorper sheep Blue: Small-tailed Han sheep FPKM: fragments per kb per transcript per million mapped reads.

The highest average expression levels of new transcripts with a size >160 bp were observed on chromosomes 1,3,10, 17 and 21 (Fig 4). Differences in average expression levels of new transcripts between SH and DP were found on chromosomes 5,10,16,17, 25 and 26. Further differential expression analysis revealed 2199 differentially expressed transcripts (absolute value of FPKM log2 Ratio≥1) between DP and SH, while 1373 were down-regulated and 826 were up-regulated in SH (S2 Table). Since the first priority of this study is to classify all of the new transcripts, both of the differentially expressed transcripts and non-differentially expressed transcripts were accepted for further analysis.

### Classification of new transcripts based on their protein-coding capacity

According to recent research, non-coding RNAs, with multiple lncRNAs, have accounted for many of the new transcripts discovered in transcriptome studies. Thus, to distinguish between coding and non-coding RNA, with less well-defined species, considerations of genome alignment-based protein-coding prediction are very important for new transcript identification in sheep [21,31].

The new transcripts were used to screen for putative coding and non-coding RNAs. To predict the protein-coding potential of new transcripts in our dataset, we applied a support vector machine-based classifier, the CPC, to assess the protein-coding potential of new transcripts based on biologically meaningful sequence features. However, it must be noted that the database used for training and the threshold for this program are somewhat arbitrary [21]. To increase the reliability of the final classification results, our dataset of 3,467 new transcripts was screened for candidate transcripts using CPC based on two separate protein databases (UniRef 90 and NCBI nr). The results were only accepted when a transcript was assigned the same protein-coding capacity prediction in both databases.

A total of 2,156 new transcripts received concordant classification (Table 1). The intersection between the prediction results based on different databases revealed 151 potential protein-coding transcripts (S3 Table) and 2,005 potential non-coding transcripts (S4 Table). Moreover, the coding potential of the other 1,311 transcripts (S5 Table) that could not achieve consistent coding potential results between different selected databases were designated as unclear.

**Table 1:** Protein-coding potential prediction of new transcripts.

**Database for analysis**	**Coding**	**Noncoding**	**Ambigious**
UniRef90	223	2201	1042
NR	410	2216	840
Intersection	151	2005	1311

### Identification of new transcripts by comparative sequence analysis

Currently, the annotation level of the reference sheep genome remains poor compared to the human and cow genomes [1,4]. To identify the transcripts already annotated in other species or related to known genes, comparative sequence analysis was executed. However, a simple similarity result obtained by sequence alignment software could not be directly adopted. Gene and pseudogene families with high similarity might introduce high false-positive results. Upon manually processing the detailed classifications, the data on current annotation status, exon structure, position on chromosome and nearest neighbor genes were also used to improve the credibility of the comparative sequence analysis.

In the results of our analysis, 246 new transcripts displayed conserved interspecies or intraspecies sequence similarities (Table 2). Among them, 30 were predicted to be protein-coding transcripts, 137 were predicted to be non-coding transcripts, and the remaining 79 transcripts could not achieve consistent coding potential results. Of the 246 new transcripts with characterization results, a total of 86 are supported by evidence from ab initio sheep gene models predicted by the Gnomon algorithm.

**Table 2:** Comparative sequence analysis result of new transcripts.

**Differential type**	**Number of transcripts**	**Number of loci**
Incompletely annotated gene	94	83
UTR	49	42
Novel gene	50	28
LncRNA	40	39
Pseudogene	2	2
Unknown protein	11	11

Based on comparative sequence analysis, conserved gene structure hypothesis, and information on neighboring gene loci, 143 of the 246 new transcripts suggest the existence of additional exons or untranslated regions for 122 sheep genes that might be incompletely annotated in the reference sheep genome assembly, and 49 show similarity to untranslated regions (UTRs), which might belong to the incompletely annotated UTRs of 42 known genes. Furthermore, 50 new transcripts may represent 28 potential new sheep gene loci not yet annotated, and 40 new transcripts were considered to belong to 39 lncRNA loci. Additionally, 2 new transcripts could be assigned to 2 pseudogene loci. The other 11 new transcripts might represent 11 unnamed protein-coding gene loci. These 246 transcripts show uneven distribution on 27 chromosomes (Fig 2). Among them, 90 differentially expressed transcripts were detected between DP and SH, while 38 were down-regulated and 52 were up-regulated in SH. The specific description of the comparative analytical results of the 246 transcripts is offered in (S6 Table).

### Classification of lncRNAs

After prediction based on protein-coding potential and comparative sequence analysis, the remaining 1867 uncharacterized new transcripts predicted to possess non-coding potential should represent putative non-coding RNAs (ncRNAs). Representing a major unexplored component of the genome [32], lncRNAs should account for a large proportion of putative ncRNAs. The functions of these lncRNAs remain largely unknown. Thus, we sought to identify a relatively comprehensive set of sheep lncRNAs from the dataset of putative ncRNAs.

The broad term lncRNA are defined as transcript >200 nt in length, does not contain a protein coding sequence, and distinguishing from short noncoding RNAs [33,34]. More than 95% of protein coding genes have ORFs of more than 100 aa [35]. In order to obtain a more reliable recognition result, only transcripts that meets the conditions of at least 200 bp in length and do not encode an ORF of more than 100 amino acids can be retained for further classification. It is also important to remove known classes of ncRNAs such as housekeeping lncRNAs and precursors of miRNA from our dataset [3,36]. In order to meet the above requirments, the lncRNA Finder was selected as our lncRNA identification pipeline.

Based on lncRNA Finder, a summary of the results is presented in Additional file 6, indicating that 315 transcripts (16.87%) were eliminated for not meeting the requirements of transcript size and ORF length. Furthermore, 17 transcripts (0.91%) showed sequence similarity to housekeeping lncRNAs, and 15 transcripts (0.80%) showed similarity to known protein domains. Finally, a total of 1,520 transcripts (81.41%) were finally considered as high-confidence lncRNA (S7 Table). A total of 1025 differentially expressed high-confidence lncRNA transcripts from 1020 loci were detected between DP and SH, while 651 were down-regulated and 374 were up-regulated in SH. Their distribution among the sheep chromosomes is described in (Fig 2).

To test the expression specificity of high-confidence lncRNA transcripts between species, a sequence similarity analysis was conducted. Based on the expressed sequence tag (EST) sequences of several species (human, mouse, rat, goat, cow and sheep), a total of 334 transcripts were specifically expressed in ruminants (S8 Table).

### Experimental validation of selected new transcripts

To confirm the accuracy of the identification result, 20 high expression transcripts were selected from different classification categories. Thus, the expression of the 20 transcripts was examined in various tissues using semi-qRT-PCR.

**Fig 5:**
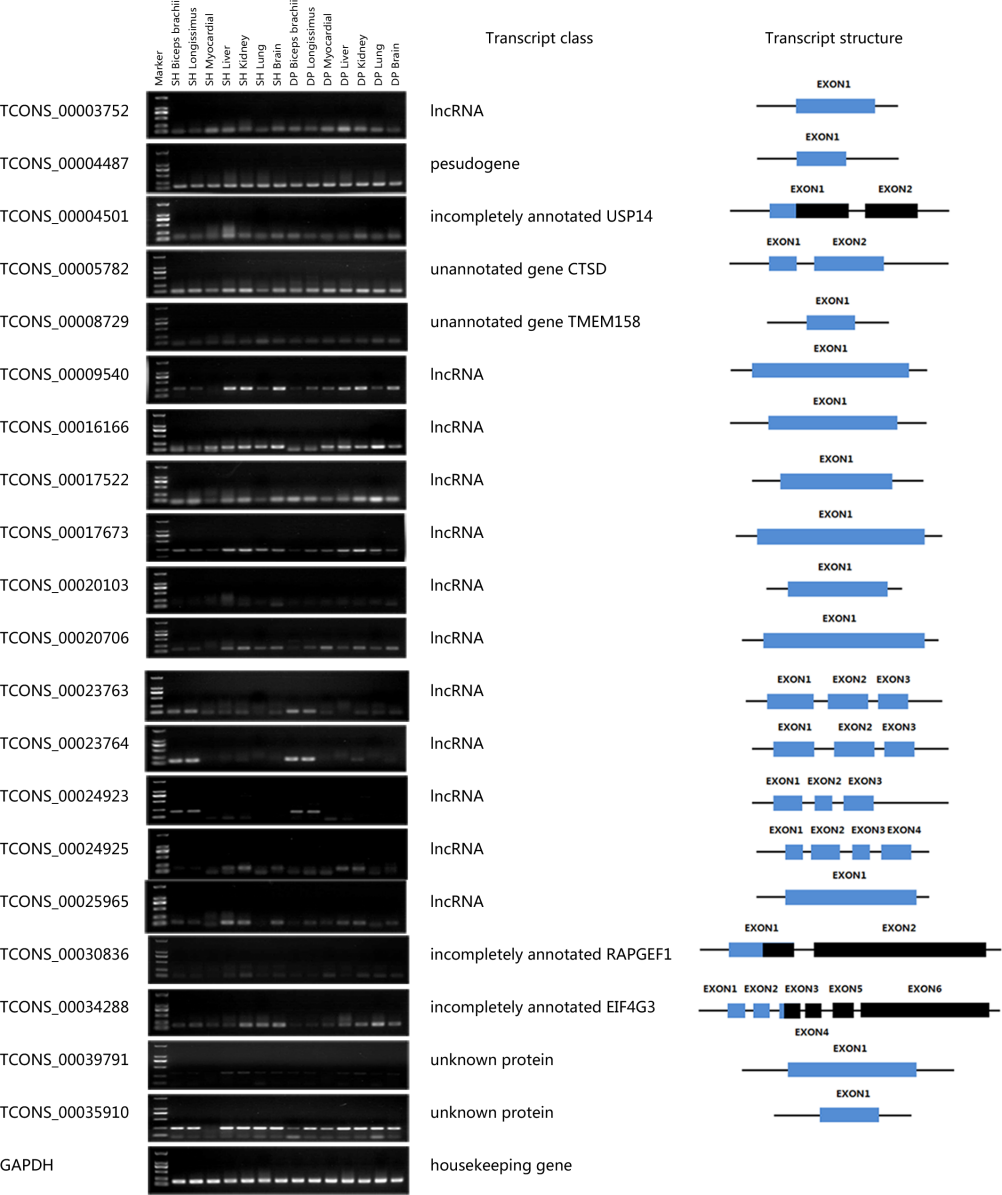
Tissue-specific expression patterns of selected new transcripts. Transcript structure is illustrated schematically: black boxes represent annotated exons (black framed: in silico predicted exons), blue boxes indicate novel exonic transcript information (blue framed box: untranslated exonic region) obtained in our study.

The exon structures of 20 selected transcripts were confirmed using the sequencing results of RT-PCR-amplified fragments (Fig 5). RNA expression profiling in a panel consisting of seven different sheep tissues revealed that 3 of the 20 transcripts are mainly expressed in skeletal muscle (Fig 5). Interestingly, all of the 3 specifically expressed transcripts were classified as lncRNA, with a detailed description of these 3 transcripts to follow.

#### XLOC_013968

The locus XLOC_013968 (consist of transcript TCONS_00023763 and TCONS_00023764) was identified as a non-coding gene on chromosome 7 and was not yet annotated in the reference sheep genome. Both of these two transcripts present differential expression between small-tailed Han sheep and Dorper sheep biceps brachii (TCONS_00023763 FPKM: 1,200.72 vs. 6,082.89; TCONS_00023764 FPKM: 1,529.59 vs. 5,443.14). The coding capacity prediction based on the two databases showed non-coding results (TCONS_00023763 Uniprot: -0.96, nr: -0.98; TCONS_00023764 Uniprot: -0.95, nr: -0.95). Comparative sequence analysis based on the NCBI nt database detected high sequence similarity with predicted *Bos taurus* non-coding locus *LOC104973263* and predicted *Bubalus bubalis* non-coding locus *LOC102407273*. It is worth noting that we found no similar sequences in the comparison with the NCBI EST database, which may indicate that TCONS_00023763 and TCONS_00023764 are specifically expressed in ruminants.

#### TCONS_00024923

The transcript TCONS_00024923 was identified as an lncRNA on chromosome 6 and was not yet annotated in the reference sheep genome. This transcript presents differential expression between small-tailed Han sheep and Dorper sheep biceps brachii (FPKM: 1,455.78 vs. 3,118.28). The coding capacity prediction based on the two databases showed a non-coding result (Uniprot: -0.96, nr: -0.62). Comparative sequence analysis based on the NCBI nt database detected high sequence similarity with predicted *Bos taurus* non-coding locus *LOC104972733*, predicted *Capra hircus* non-coding locus LOC106502188 and predicted *Bison bison bison* non-coding locus LOC104988555. We believe that transcript TCONS_00023763 is specifically expressed in ruminants, supported by the sequences of NCBI EST database. Furthermore, according to the result of expression profile (Fig 5), TCONS_00024923 might be specifically expressed in skeletal muscle tissue.

## Discussion

In our previous report, to understand the factors influencing muscle growth in sheep with different growth rates, Dorper and small-tailed Han sheep with the same age and different growth rates were selected as research subjects [15,16]. Information on differentially expressed genes, AS and cSNPs were reported after a detailed study, and new transcript units were also reported as part of our past studies [15,16]. However, without detailed annotation and classification, we could not conduct further study of the new transcripts. Moreover, due to the frequent updates of the sheep reference genome and gene annotation data, our past results have been unable to serve as a reference for further research. For these reasons, we decided to reanalyze the sequencing data based on the latest genome annotation to reveal the new transcripts.

Consistently with transcriptome-wide studies in other species [2,3,4,37], the results of our research have also revealed a large number of new transcripts. Ultimately, 40,129 unique primary muscle transcripts were assigned to the sheep genome. Among them, 3,467 transcripts (8.64%) were not annotated in the reference sheep genome, a slightly lower proportion than in our previous report [15,16] and in similar research on sheep skin [11]. This result is believed to be due to the continuous improvement of the genome information, the complementarity of known gene structure and the annotation of new gene loci. In addition, the new transcripts were distributed evenly among the chromosomes. These distribution characteristics indicate that the new transcripts are not a product of transcriptional noise.

Of the 3,467 new transcripts, a total of 246 have been clearly identified by comparative sequence analysis. Such results could be an effective supplement to reference genome annotation. It is worth noting that among the 246 identified transcripts, 11 protein-coding transcripts were determined to be unknown proteins because they did not match up with any functional annotated genes. This result indicates that there are still many proteins of unknown function that play roles in skeletal muscle growth. The functions of these unknown proteins require further study.

Our analysis identified a number of reliable potential lncRNAs. This dataset of 1,520 lncRNAs should be useful for sheep skeletal muscle regulation research or in the study of possible functional differences among sheep varieties. Interestingly, using the same method as Li and Eichten [3], we found no transcripts containing homologous sequences to miRNAs, even though miRNA precursors should be important components of putative lncRNAs [7,34]. This result might be due to the poor annotation level of sheep miRNA compared to other species, such as human and cow; that is, the total number of annotated sheep miRNAs (153) supported by miRBase is far less than the numbers in human (2,588) and cow (793). Using the same method as described above, a small amount of acceptable alignment results were detected during the comparison with human (18 hits) and cow (60 hits), which could be an evidence for our hypothesis. This result also means that a group of small RNA precursors should be included in our dataset of high confidence lncRNAs. Compared to protein-coding genes, lncRNAs are more likely to show interspecific specificity [38]. The recognition of lncRNAs with specific expression characteristics should be useful to help explain the regulation of gene expression and to improve the reference genome annotation. To the best of our knowledge, no transcriptome-wide lncRNA recognition had previously been executed in sheep skeletal muscle. Supported by EST data on several species, 334 lncRNA transcripts were specifically expressed in ruminants. Moreover, among them, 3 lncRNA transcripts were found to be specifically expressed in sheep skeletal muscle, which indicates they might play important roles in the regulatory processes of sheep skeletal muscle. Further research will be performed to confirm the specific functions of these transcripts.

A total of 2199 new transcripts were identified as differentially expressed transcripts, of which 1115 were eventually got the classification results. Compared to our previous report [16], these transcripts revealed the unknown portion of the difference between the two transcriptome, which greatly improved our understanding of the sheep skeletal muscle transcriptome complexity. However, only 90 of them received comparative sequence analysis result. As for the other 1025 differentially expressed transcripts which were classified as high-confidence lncRNA, it is still unable to confirm their physiological function. To identify the small RNA precursors and competitive endogenous RNA from high-confidence lncRNA, a sufficient credible sheep small RNA dataset should be greatly helpful, and this should also be the focus of our future research.

## Conclusions

In this study, a catalogue of new transcripts for sheep skeletal muscle was generated based on a whole-transcriptome RNAseq approach. A complex transcript pattern in sheep skeletal muscle, including protein-coding and non-coding RNA, was verified. A total of 3,467 transcripts were not annotated in the reference sheep genome and were defined as new transcripts. A total of 246 new transcripts were classified as portions of unannotated or incompletely annotated genes, and 1,520 transcripts were predicted with high confidence to be lncRNAs. Furthermore, our analysis revealed 334 new lncRNA transcripts that displayed specific expression in ruminants. The expression of 20 selected new transcripts was confirmed in several sheep tissues by RT-PCR, 3 lncRNA transcripts without intergenus homology but with specific expression in sheep skeletal muscle were also been discovered. The results suggest that new transcripts without annotations still occupied a considerable proportion of the sheep transcriptome. These novel transcripts should not be ignored in transcriptome and genomic studies, as they might play important roles in regulation. Collectively, our results complement the current sheep reference genome annotation and constitute a valuable resource for the identification of new transcripts underlying the muscle growth of sheep, which might aid future studies.

## Supporting Information

S1 Fig Transcriptome annotation level comparison. (A) Total reads mapping rate comparison. (B) Specifically expressed and co-expressed gene loci number comparison. (C) New transcript number comparison.
(TIF)

S1 Table Primer sequences for RT-PCR. (XLS)

S2 Table Unannotate new transcripts. (XLS)

S3 Table Potential protein-coding transcripts. (XLSX)

S4 Table Potential non-coding transcripts. (XLSX)

S5 Table Potential unclear transcripts. (XLSX)

S6 Table Comparative analytical result. (XLSX)

S7 Table High confidence lncRNA. (XLSX)

S8 Table Specifically expressed in ruminants. (XLS)
