# Supplementary figures and images for: Identification and Classification of New Transcripts in Dorper and Small-Tailed Han Sheep Skeletal Muscle Transcriptomes
Source: PLoS One. 2016 Jul 19;11(7):e0159638. doi: 10.1371/journal.pone.0159638 (PMC4951087; doi:10.1371/journal.pone.0159638)

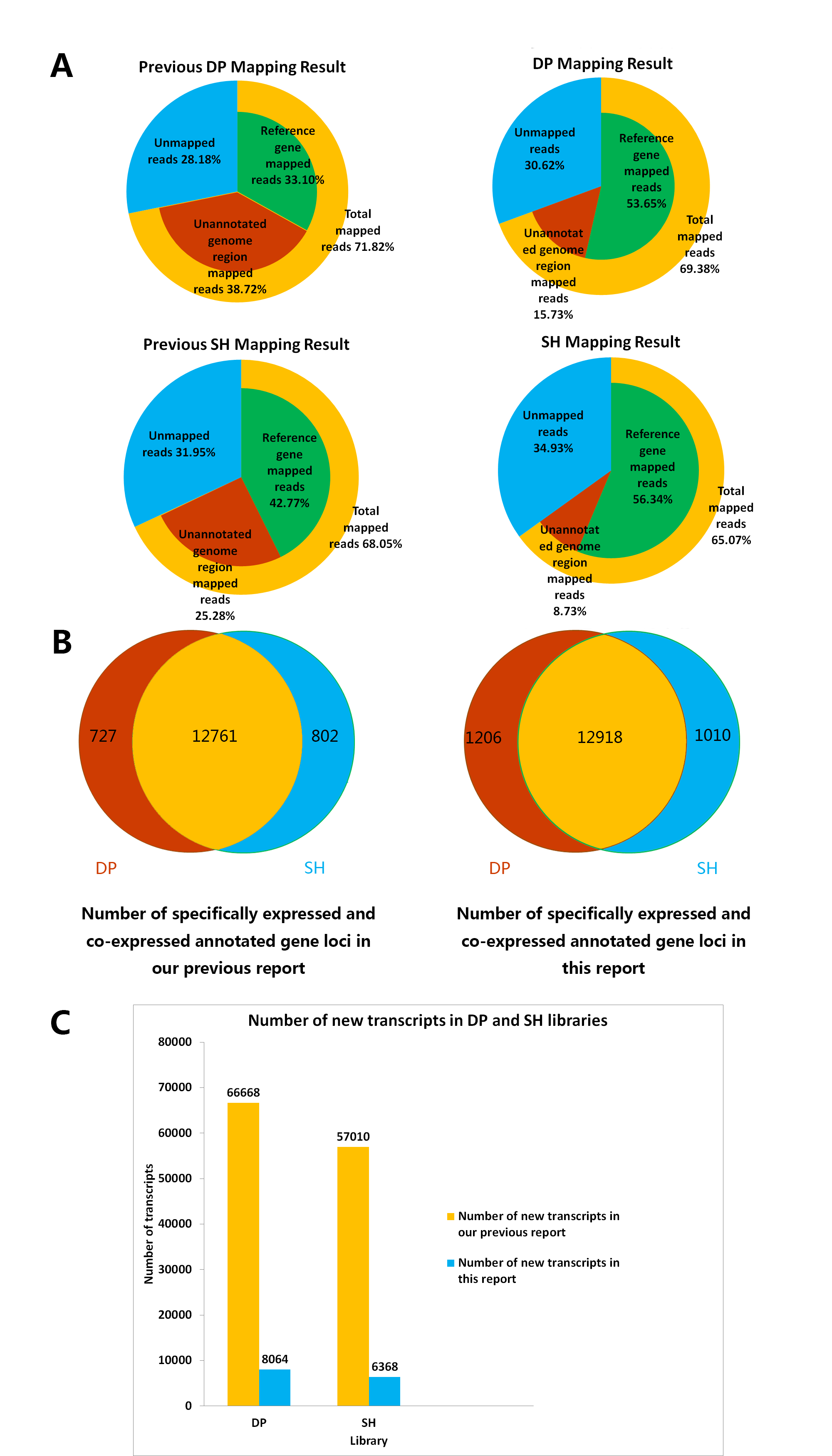

Supplement: S1 Fig — (A) Total reads mapping rate comparison. (B) Specifically expressed and co-expressed gene loci number comparison. (C) New transcript number comparison. (TIF) [file pone.0159638.s001.tif]
